# Impact of the introduction of a package of care involving early detection of opportunistic infections, a prospective multicenter cohort study of people living with HIV/AIDS in Brazil

**DOI:** 10.1016/j.lana.2025.101085

**Published:** 2025-04-03

**Authors:** Alessandro C. Pasqualotto, Omar Sued, Nicole Reis, Larissa R. Silva, Renata B.A. Soares, Cassia S.M. Godoy, Marineide G. Melo, Nayla A. Hatem, Bruna Regis Razzolini, Andressa Noal, Tarsila Vieceli, Diego R. Falci, Freddy Perez

**Affiliations:** a Federal University of Health Sciences of Porto Alegre, R. Sarmento Leite, 245 - Centro Histórico, Porto Alegre, RS, 90050-170, Brazil; b Santa Casa de Porto Alegre, Porto Alegre, RS, Brazil; c Pan American Health Organization (PAHO), 525 23 ST NW, Washington DC, 20037, USA; d Hospital Vila Nova, R. Catarino Andreatta, 155 - Bairro Vila Nova, Porto Alegre, RS, 91750-040, Brazil; e Hospital Estadual de Doenças Tropicais Dr Anuar Auad, Alameda do Contorno, 3556, Jardim Bela Vista, Goiânia, GO, 74850-400, Brazil; f Pontifícia Universidade Católica de Goiás, Goiânia, GO, Brazil; g Grupo Hospitalar Conceição, Av. Francisco Trein, 596 - Cristo Redentor, Porto Alegre, RS, 91350-200, Brazil; h Hospital de Clínicas de Porto Alegre, Rua Ramiro Barcelos, 2350 Bloco A, Av. Protásio Alves, 211 - Bloco B e C - Santa Cecília, Porto Alegre, RS, 90035-903, Brazil; i Pontifícia Universidade Católica do Rio Grande do Sul, Porto Alegre, RS, Brazil

**Keywords:** Brazil, Cohort study, HIV, AIDS, Tuberculosis, Histoplasmosis, Cryptococcosis, Point-of-care testing

## Abstract

**Background:**

Opportunistic infections (OIs) significantly contribute to morbidity and mortality in advanced HIV disease. This study evaluates the efficacy of point-of-care (POC) diagnostics for tuberculosis (TB), histoplasmosis, and cryptococcosis in routine HIV care in Brazil.

**Methods:**

A prospective multicenter cohort study was conducted across five hospitals enrolling people living with HIV (PLHIV) with CD4+ T-cell count <200 cells/mm^3^ or OI symptoms, regardless of CD4 count, HIV-naïve patients, those initiating treatment, and individuals with unsuppressed viral load lost to follow-up (>3 months). POC tests included VISITECT CD4 Advanced Disease, TB LAM Ag (Abbott), GeneXpert MTB/RIF (Cepheid), Histoplasma antigen LFA (MiraVista), and CrAg LFA (IMMY). Patients were followed at 30 and 90 days. Retrospective data for six months pre-study was collected for comparison.

**Findings:**

Among 419 PLHIV (55% cisgender men, 44% cisgender women, 1% transgender; mean age: 42 years, SD ± 11.1), 46% had confirmed OIs: TB (34%), cryptococcosis (12%), histoplasmosis (10%). Co-infections were frequent, with TB and histoplasmosis (44%). Cryptococcal meningitis and severe histoplasmosis were diagnosed in 5% and 6%, respectively. TB LAM was positive in 27% of tested patients, with 74% having disseminated TB. POC testing increased detection rates for TB, (1.8-fold) cryptococcosis (2.8-fold), and histoplasmosis (2.8-fold) compared to historical data. Survival rates were 87% at 30 days and 80% at 90 days, with cryptococcal antigenemia associated with higher mortality.

**Interpretation:**

POC testing improved OI diagnosis, aligning with WHO guidelines. These findings highlight the importance of integrating rapid diagnostics into HIV programs and the need for further research on long-term outcomes.

**Funding:**

10.13039/100011893Pan American Health Organization.

**Research in context**

**Evidence before this study**

Opportunistic infections (OIs) remain a leading cause of HIV-related mortality, despite the widespread rollout of antiretroviral therapy. However, the exact burden of HIV-related OI is uncertain due to underdiagnosis in routine care. National health policymakers in Latin America require further evidence to support the broader implementation of point-of-care (POC) testing for HIV-related OIs. We searched PubMed and Google Scholar from January 2018 to March 30, 2024, using the terms “*HIV advanced* *disease*”, “*opportunistic infections*”, “*diagnosis*”, “*rapid tests*”, and “*mortality*”. No language restrictions were applied. Few cohort studies have demonstrated the prevalence and impact on mortality of OIs among people living with HIV (PLHIV) using rapid routine screening for opportunistic infections. Notably, in Guatemala, POC testing for OIs reduced overall mortality associated with these conditions in PLHIV. Similarly, a study in Central America indicated that the implementation of rapid diagnostic assays increased the detection of histoplasmosis and cryptococcosis in PLHIV. However, no studies were found that compared retrospective data to assess the potential impact of introducing POC diagnostic tests for HIV-related OIs.

**Added value of this study**

Our study provides new evidence on the value of POC testing in enhancing the diagnostic capacity for histoplasmosis, cryptococcosis, and tuberculosis (TB) among patients with advanced HIV disease (AHD). This was achieved through a prospective multicenter cohort study conducted across five tertiary care centers in Brazil. Participants were followed for 30-and 90-days. Additionally, we provide insights from retrospective data collected over the preceding six months for comparative analysis. Our findings underscore opportunities for improving care for PLHIV.

**Implications of all the available evidence**

These findings demonstrate the added value of integrating POC tests for the early diagnosis of TB, cryptococcosis, and histoplasmosis in hospitalized patients with AHD in Brazil. This integration led to a substantial increase in OI diagnoses, facilitating the management of these complex medical conditions. Scaling up this intervention has the potential to significantly reduce HIV-related mortality in routine care. However, this requires ensuring the availability of POC tests and access to treatment for these OIs to mitigate HIV-associated mortality. The results of this demonstration project offer evidence that may inform the optimization of national policies and updates of national guidelines for PLHIV with advanced HIV disease.

## Introduction

Despite significant advancements in antiretroviral therapy (ART), advanced HIV disease (AHD) remains a challenge.^1^ In many settings, limited access to testing, delays in diagnosis, and barriers within the healthcare systems contribute to the persistence of AHD. Additionally, patient retention within the healthcare system is difficult, with many individuals lost to follow-up during routine care, further impacting patient outcomes. These systemic issues highlight the need for improved healthcare infrastructure and expanded access to early HIV diagnosis and treatment.

Several strategies have been proposed to reduce the morbidity and mortality associated with AHD.^2^ The World Health Organization (WHO) recommends a package of interventions for this patient population,^3^ including screening for opportunistic infections (OIs), prophylaxis, and prompt initiation of ART along with intensified adherence support. In this context, early diagnosis of OIs is a key factor for proper disease management. In Guatemala, screening for OIs using point-of-care tests has reduced the overall mortality associated with these conditions in people living with HIV/AIDS (PLHIV).^4^ With the introduction of lateral flow immunochromatography tests, a fast, reliable, and point-of-care diagnosis is now possible for the most important OIs affecting PLHIV, such as tuberculosis (TB), cryptococcosis, and histoplasmosis.^5^, ^6^, ^7^

Brazil has one of the highest HIV burdens in Latin America,^8^ and is recognized for its comprehensive HIV/AIDS response. The Brazilian public healthcare system (Sistema Único de Saúde, or SUS) provides free access to HIV testing and ART, allowing broader accessibility to diagnosis and treatment.^9^ This policy of free HIV testing and treatment has significantly contributed to reducing HIV-related morbidity and mortality rates in Brazil.^10^, ^11^, ^12^, ^13^, ^14^ However, significant challenges still remain in the country, including concentrated burden of disease in high-vulnerability groups, inequality of care and sustainability of the HIV program.^15^^,^^16^ Experience is still limited in terms of tools for rapid diagnostics of OIs in the advanced HIV population in Brazil, and routine screening for some (i.e., histoplasmosis) is not yet standard practice. This study aims to assess the efficacy of rapid routine screening for TB, cryptococcosis and histoplasmosis among symptomatic and advanced HIV patients in five hospitals in Brazil. We hypothesize that implementing rapid routine screening for these conditions is feasible and could lead to better clinical outcomes in Brazil.

## Methods

### Sites and participants

This prospective multicenter cohort study was conducted in five tertiary reference medical centers for HIV care in Brazil. Four centers are located in Porto Alegre, Southern Brazil: Associação Hospitalar Vila Nova, Grupo Hospitalar Conceição, Hospital de Clínicas de Porto Alegre, and Santa Casa de Porto Alegre. The fifth center is in Goiânia, Central-West Brazil: Hospital Estadual de Doenças Tropicias Dr Anuar Auad. These centers were selected due to the high HIV prevalence in these settings, and their prior experience in clinical and epidemiological studies.

Between January and December 2023, all adult (>18 years-old) HIV patients admitted to any of these institutions were considered for participation. Inclusion criteria included: (i) patients with AHD, defined by a confirmed HIV infection and: a) CD4 count <200 cells/mm^3^ (test performed at study entrance or within the last 3 months); or b) the presence of signs/symptoms suggesting the presence of an OI (including fever, cough, weight loss, night sweats, abnormal mental state, headache, focal neurological signs, lymphadenopathy or cutaneous/mucosal lesions), regardless of the CD4 count (asymptomatic patients were not studied); (ii) not receiving sustained and effective ART, defined as a) HIV naïve patients without treatment; b) ART initiated within the last 3 months; c) loss of follow-up while on treatment (>3 months) with a unsuppressed viral load; or d) virological failure (two consecutive detectable HIV viral loads, with at least one viral load being >1000 copies/ml). Patients were excluded if they had received active therapy for both TB and systemic fungal diseases in the last two weeks.

### Ethics statement

Participation was voluntary and required signed informed consent. The study was based on a master protocol approved by the PAHO Ethical Committee (PAHOERC) under register PAHOERC.0347.01, as well as by the ethical committees of all participant institutions. No personal identifiable information was collected in the database used for the analysis. Data was compiled by trained professionals and stored in a secure database.

### Procedures

The primary aim was to assess the efficacy of implementing a novel model of care for individuals with AHD. This involved introducing a comprehensive package of diagnostic assays to facilitate early diagnosis and treatment for cryptococcosis, histoplasmosis and TB in PLHIV, with the potential to reduce overall mortality. Secondary endpoints included determining the prevalence of these infections in the study population.

Demographic data, including sex, gender, age, race/ethnicity, years of education, and family income were collected for all study participants. Clinical data, including previous OIs, use of recreational illicit drugs (i.e., other than alcohol, tobacco, and medicines), and signs/symptoms, were also documented. Information related to HIV infection comprised date of diagnosis, the latest CD4 count, HIV viral load results, use of ART, and recent (within the past 14 days) use of antitubercular and antifungal drugs.

Upon enrollment, patients underwent evaluation using a rapid CD4 count lateral flow assay (Accubio VISITECT CD4 Advanced Disease Lateral Flow Assay; Accubio Limited, Scotland, UK), unless a recent (<3 months) CD4 count was available. In case of a discordant result, CD4 quantification by flow cytometry was used for final reporting. All laboratory personnel involved in reading point-of-care test results received training from a central team. TB screening was performed using the TB LAM Ag testing (Alere Determine TM TB LAM Ag; Abbott, Palatine, IL, USA) on urine samples. Centers were encouraged to investigate active TB in patients with respiratory symptoms using a molecular test (Xpert® MTB/Rif; Cepheid, Sunnyvale, CA, USA).

TB was diagnosed in patients who were smear- or culture-positive, or who had a positive molecular or TB LAM result. Patients were further classified as having disseminated TB if cultures or molecular tests for TB were positive at extra-pulmonary sites. Disseminated TB was diagnosed if the TB LAM was positive in the absence of confirmed pulmonary TB by microscopy, culture and/or molecular test). Although all research personnel were trained to perform point of care testing, there was no specific recommendation regarding which team members should conduct the tests at individual sites.

Cryptococcosis screening was performed using cryptococcal antigen detection in serum (CrAg® lateral flow assay; IMMY, Norman, OK, USA). Evaluation for central nervous system infection was recommended for all patients who tested positive for CrAg in serum. Patients with positive CrAg in serum were classified as having cryptococcal disease. Lumbar puncture was recommended for all patients with cryptococcal antigenemia. Meningoencephalitis was defined by either a positive culture of *Cryptococcus* species or a positive CrAg result in cerebrospinal fluid. Extra-meningeal cryptococcosis was defined as a positive CrAg in serum or a positive extra meningeal culture of *Cryptococcus* species in the presence of disease with no central nervous system involvement.

Histoplasmosis screening was conducted using *Histoplasma* urine antigen lateral flow assay (MiraVista, Indianapolis, IN, USA). Histoplasmosis was diagnosed based on a positive *Histoplasma* antigen result in urine or other laboratory evidence such as positive microscopy, histopathology, or culture. Patients with a positive *Histoplasma* antigen were classified according to PAHO/WHO guidelines as having mild/moderate histoplasmosis or severe disease.^17^

Patients were followed at 30- and 90-day post-enrollment to assess overall survival and the occurrence of severe adverse events. These timeframes were selected based on prior studies in AHD which have shown that early mortality (within the first 30 days) is often associated with undiagnosed or untreated opportunistic infections (OIs),^18^, ^19^, ^20^, ^21^ while 90-day outcomes provide a more comprehensive assessment of the impact of early diagnosis and treatment on survival. Additionally, these timeframes align with the natural history of OIs in advanced HIV, where early intervention is critical to reducing mortality.

Survival was determined through either personal visits or phone calls. Centers were asked to collect retrospective data for patients admitted six months prior to the prospective study. This approach aimed to have a potential comparison of the frequency of TB, cryptococcosis and histoplasmosis between the retrospective and the prospective phases thereby assessing the impact of introducing diagnostic tests on the prevalence of these conditions, the frequency of empirical treatment, and overall mortality.

Data was collected using a standardized form built on REDCap electronic data capture tools hosted at the Santa Casa de Porto Alegre server.^22^ Periodic data cleaning was performed centrally by the coordinating center to identify missing data and incongruences.

### Statistical considerations

Sample size was calculated using OpenEpi version 3 (https://www.openepi.com), based on an estimated prevalence of 8% for histoplasmosis^23^ (the disease with the lowest frequency among investigated OIs in the study). Considering a margin of error of 3%, and a 95%, confidence interval (CI) a sample size of 315 patients was required for the study (a minimum of 350 patients would provide a 95% CI of a prevalence of histoplasmosis between 5 and 11%). Patients from the different sites were included in the cohort as per convenience. Descriptive statistics were used to summarize the data, including measures such as means, median, standard deviation, or interquartile range. Pearson's chi-square and Fisher's exact test were employed to evaluate the association between qualitative variables, while the Mann–Whitney test was used for the comparison of quantitative variables (unadjusted association). The two-sided p-value level of significance was set at 5%. Data analysis was performed with IBM® SPSS® Statistics version 22.0.

### Role of the funding source

OS and FP participated in the study design, data analysis, data interpretation, and approved the final version of the report.

## Results

A total of 419 patients were included in the study, comprising 230/419 cisgender men (54.9%), 183/419 cisgender women (43.7%), and 6/419 transgender women (1.4%). The mean age was 42.7 years (standard deviation, +11.1 years). Patients self-identified as Brown/Pardo (141/419, 33.7%), Black (123/419, 29.4%), White (151/419, 36.0%), Yellow (3/419, 0.7%), or as Indigenous people (1/419, 0.2%) (Table 1). Educational attainment varied, with a large number of patients not completing elementary school (190/419, 45.3%), while 80/419 (19.1%) completed elementary studies, 28/419 (6.7%) had incomplete middle school, 80/419 (19.1%) had finished middle school, and 41/419 (9.8%) had achieved higher educational levels. The median monthly household income was 209.4 (Interquartile Range, IQR, 209.4–349.0) with 46.5% (195/419) reporting the use of illicit drugs.

**Table 1:** Distribution of demographic data and frequency of main opportunistic infections before (retrospective study—previous six months) and after (prospective) the introduction of point-of-care diagnostic tests.^a^

Variable	Retrospective study (n = 527)	Prospective study (n = 419)	p value
Age (years)			
Mean, ±standard deviation	44.7 (±12.6)	42.7 (±11.1)	0.015
Gender			
Male cisgender	306/527 (58.1%)	230/419 (54.9%)	0.328
Ethnicity/Racial Identity			
White	N/A	151/419 (36.0%)	
Brown/Pardo		141/419 (33.7%)	
Black		123/419 (29.4%)	
Yellow		3/419 (0.7%)	
Indigenous people		1/419 (0.2%)	
HIV infection			
Naive	149/525 (28.4%)	133/419 (31.8%)	0.262
CD4 count, median (IQR)	122 (47–301)	66 (21–156)	<0.001
Tuberculosis			
Empirical treatment	37/527 (7.0%)	83/419 (19.8%)	<0.001
Confirmed cases	94/527 (17.8%)	143/419 (33.7%)	<0.001
Cryptococcosis			
Central nervous system	12/526 (2.3%)	20/419 (4.8%)	<0.001
Disseminated	15/535 (2.8%)	32/419 (7.6%)	<0.001
Histoplasmosis			
Empirical treatment	6/527 (1.1%)	N/A	
Confirmed cases	19/527 (3.6%)	41/406 (10.1%)	<0.001
Overall survival			
30 days	433/522 (82.9%)	343/394 (87.1%)	0.088
90 days	385/513 (75.0%)	306/382 (80.1%)	0.075

Legend: IQR, Interquartile range; N/A, Data not available.

a This comparison has limitations, including potential differences in patient characteristics and data collection methods, which may influence the assessment of the impact of introducing diagnostic tests on the prevalence of the opportunistic infections and overall mortality.

Most patients were known to be HIV infected before entering the study (286/419, 68.2%), and 398 of these individuals (95.1%) had been exposed to ART. Therefore 31.8% of the study population was ART-naïve (133/419). The CD4 count was below 200 cells/mm^3^ for 95.4% of patients (400/419) with a median CD4 count was 66 cells/mm^3^ (IQR, 21–156 cells/mm^3^). The median HIV viral load was 104,887 copies/mL (5.02 log; IQR, 3.43–5.74 log). Previous opportunistic infections had occurred in 52.7% of participants (221/419), including TB (118/419, 28.2%), toxoplasmosis (83/419, 19.8%), cryptococcosis (14/419, 3.3%), and histoplasmosis (9/419, 2.1%). The median duration of HIV infection was 6.0 years (IQR, 0–12 years), from first diagnosis to study entrance. All patients were symptomatic at study inclusion, presenting with signs/symptoms suggestive of OI.

A comparison of the baseline characteristics for patients included in the retrospective (i.e., before the introduction of point-of-care tests for OIs) and prospective phase of the study is presented in Table 1. Baseline characteristics were similar during these study periods, although patients in the prospective study had more AHD, as reflected by lower CD4 counts, compared to those admitted during the retrospective phase. Importantly, the introduction of point-of-care diagnostic tests was associated with a marked increase in the diagnosis of TB, cryptococcosis, and histoplasmosis.

During the study, 191 of 419 individuals (46%) were diagnosed with one of the OIs of interest, including TB (34%, 143/419; 95% CI, 30–39%), cryptococcosis (12%, 52/419; 95% CI, 9–16%), and histoplasmosis (10%, 41/419; 95% CI, 7–13%). Table 2 shows the frequency of these OIs according to patients’ immune status (CD4 counts). Additionally, 83 of 419 patients (20%) were empirically treated for TB, without microbiological confirmation.

**Table 2:** Frequency of tuberculosis, cryptococcosis, and histoplasmosis in a cohort study of 419 patients living with HIV/aids in Brazil in a context in which point-of-care tests for opportunistic infections are offered, according to the CD4 count^a^.

	Total	CD4 count (cells/mm^3^)	
		≤200	>200
Tuberculosis			
Pulmonary	46/387 (11.9%)	41/352 (11.6%)	5/35 (14.3%)
Disseminated	85/387 (22.0%)	80/352 (22.7%)	5/35 (14.3%)
Cryptococcosis			
Meningoencephalitis	19/388 (4.9%)	18/352 (5.1%)	1/36 (2.8%)
Disseminated	29/388 (7.5%)	25/352 (7.1%)	4/36 (11.1%)
Histoplasmosis			
Mild/moderate	10/387 (2.6%)	9/352 (2.6%)	1/35 (2.9%)
Severe	26/387 (6.7%)	25/352 (7.1%)	1/35 (2.9%)

a Denominators differ based on coverage of point-of-care testing for each opportunistic infection.

Point of care tests were positive in 157 of 189 infections (83%; 95% CI, 78–88%), including TB (81%, 106 out of 131 tested; 95% CI, 74–88%), cryptococcosis (94%; 47 out of 50 tested; 95% CI 87–100%), and histoplasmosis (98%, 40/41; 95% CI, 93–100%). At baseline, the TB LAM was positive for 106/399 patients (27% of tested patients), while 293/399 (74%) tested negative (20 patients were not tested). Among those positive for TB LAM, 28 of 106 (26%) had pulmonary TB, and 78 of 106 (74%) had disseminated TB. TB treatment was initiated in 79% (83 out of 105 tested) of patients with a positive TB LAM result, compared to 19% (54 out of 291 tested) of those with negative TB LAM result (p < 0.001). TB LAM was positive in 62% of patients with confirmed pulmonary TB (28 of 45). Disseminated TB occurred in 86 patients, 91% (78 of 86 tested) had a positive TB LAM test, with 11 patients having a positive molecular test for TB in a non-respiratory sample. Among patients with positive TB LAM results (n = 106), 31% (n = 33) were not evaluated with neither GeneXpert® MTB/RIF or sputum smear microscopy.

In the screening for cryptococcosis, serum CrAg was positive in 47 of 406 patients (12%; 95% CI, 9–15%). Cryptococcal meningoencephalitis was diagnosed in 20 patients (5% of the total and 43% of the positive CrAg cases). Extra-meningeal cryptococcosis was identified in 32 patients (8%). Lumbar puncture was not performed in 2 of the 47 patients (4%) with positive serum CrAg. Moreover, CrAg testing in cerebrospinal fluid was not requested in 9 of the 45 patients (20%) who had a lumbar puncture performed after testing positive for serum CrAg, due to protocol violations.

*Histoplasma* urine antigen was positive in 41/405 patients (10%; 95% CI, 7–13%). Among these, 26 patients had severe histoplasmosis (all of whom tested positive for *Histoplasma* antigenuria), and 15 patients had mild or moderate histoplasmosis (9 of 10 tested positive for *Histoplasma* antigenuria). *Histoplasma* culture was positive in seven patients, including one case with negative antigenuria. Severe histoplasmosis cases occurred almost exclusively in patients with CD4 counts of <200 cells/mm^3^ (median 31 cells/mm^3^; IQR, 3–231 cells/mm^3^). Certain tests (13 CrAg and 14 *Histoplasma* urine antigen) were not performed due to previous antifungal treatment within the last 14 days, unavailability of test diluent, or urine collection difficulties, such as early discharge and mortality. Among histoplasmosis cases, 49% were reported in the city of Goiania.

Several co-infections of two or more OIs were also observed. Sixteen patients (4%, 16/390; 95% CI, 2–6%) tested positive for both TB LAM and CrAg. Additionally, 18 patients were co-infected with TB and *Histoplasma* (5%, 18/392; 95% CI, 3–7%), accounting for 44% of histoplasmosis cases. Seven patients had concurrent TB, cryptococcosis and histoplasmosis.

On day 30 of follow-up, 343 out of 394 patients (87%; 95% CI, 84–90%) were still alive, and 25 out of 419 were lost to follow-up (6.0%). By day 90, survival had decreased to 80% (306 out of 382; 95% CI, 76–84%), with 37 out of 419 patients lost to follow-up (9%). As shown in Table 3, positivity for point-of-care tests for TB and histoplasmosis had no impact on overall patient survival. In contrast, a positive antigen test for *Cryptococcus* species was associated with an increased overall mortality at both 30 and 90 days of follow-up.

**Table 3:** Survival impact of point-of-care positivity for different opportunistic infections in people living with HIV/AIDS.

Test result	Survival at day 30	p value	Survival at day 90	p value
TB LAM				
Positive	86.9% (86/99)	0.899	81.6% (80/98)	0.693
Negative	87.4% (242/277)		79.8% (213/267)	
Cryptococcal antigen				
Positive	69.6% (32/46)	<0.001	66.0% (31/47)	0.010
Negative	89.3% (300/336)		82.1% (266/324)	
*Histoplasma* antigen				
Positive	77.5% (31/40)	0.054	75.0% (30/40)	0.370
Negative	88.3% (301/341)		81.0% (268/331)	

## Discussion

This study demonstrates the added value of diagnostic tests for rapid diagnosis of common OIs affecting PLHIV. Nearly 45% of patients had a confirmed OI and an additional 20% received empirical treatment. One third (34%) of all the patients were diagnosed with TB; most of whom tested positive for TB LAM. It was no surprise to observe TB as the primary OI in patients with advanced HIV infection in this study, consistent with previous studies conducted in Brazil, given the severely compromised immune status of these patients, as indicated by their low CD4 counts.^24^ Additionally, 13% of patients (n = 35) received empirical therapy for TB without microbiological confirmation or a positive TB-LAM test, a practice justified by the high TB prevalence in this population. However, there is a need to improve the coverage of laboratory-confirmed TB and the prompt diagnosis for other infections.^25^

Despite the high frequency of TB, our data reveals areas for improvement in patient's care. Notably, 31% of patients with positive TB LAM results did not undergo molecular tests on respiratory samples and the same percentage did not have sputum bacilloscopy. This raises concerns about underestimating the prevalence of pulmonary TB, a highly contagious disease requiring appropriate isolation precautions. Alternatively, some false-positive TB LAM results may have contributed to the high positivity rate.^26^, ^27^, ^28^ Additionally, it is worth noting that nearly a third (32%) of patients entering this study were not aware of their HIV status before hospital admission, emphasizing the need for expanding HIV testing in the general community to enhance epidemic control.

Cryptococcosis and histoplasmosis were also frequently observed in this study accounting for 12% and 10% of diagnosis respectively. Regional disparities were noted in the frequency of histoplasmosis, with 49% of cases occurring in the Central-West region of Brazil (Goiânia), a known endemic area for histoplasmosis.^29^

Co-infection of histoplasmosis with TB was high (44%, 18/41 histoplasmosis cases), particularly when compared to both the general HIV population and the HIV-negative population. Previous studies in Latin America reported frequencies ranging from 2.0% to 38.0%, depending on the stage of immunosuppression and diagnostic methods used.^30^ This co-infection poses a significant treatment challenge, as itraconazole and rifampicin, drugs used for histoplasmosis and TB respectively have an important drug–drug interaction, reducing itraconazole levels when co-administered with rifampin. Frequent treatment failures have been documented, and no optimal strategy exists for managing the maintenance phase of histoplasmosis in these cases. Itraconazole levels should be monitored.^17^ Furthermore, recent clinical trials have excluded patients with TB and histoplasmosis.^31^ There is an urgent need to clarify the best strategy to effectively treat histoplasmosis and TB co-infection in a way that is feasible for public health.

Overall, the introduction of point-of-care diagnostic tests improved the diagnostic capacity for the most frequent OIs in this study, providing a significant incremental benefit in addition to traditional diagnostic methods. This cohort study conducted in five HIV reference centers in Brazil, focused on patients with advanced HIV. The demographic profile of the study population was predominately young poor, male, individuals of Brown (Pardo) or Black ethnicity, with low educational levels and limited adherence to ART. Our data clearly demonstrates the significant impact of rapid diagnostic tests for OIs in improving patient care within the advanced HIV care cascade, surpassing retrospective data in diagnosing OIs. This is particularly crucial in a population characterized by lower socio-economic status and educational levels, where barriers to healthcare access and adherence to treatment regimens are more pronounced. For instance, rapid tests increased the detection of TB, cryptococcosis, and histoplasmosis by 1.8-fold, 2.8-fold, and 2.8-fold, retrospectively, compared to historical series. Although this had limited impact on overall patients’ survival—except for cryptococcal antigen detection—the impact on patient care in noteworthy. However, these comparison limitations, including potential differences in patient characteristics and data collection methods could influence the assessment of the impact of introducing diagnostic tests on the prevalence of these conditions and overall mortality.

Our findings advocate for the integration of point-of-care testing into routine clinical practice within the Brazilian HIV/AIDS program. In support of this, all the rapid diagnostic tests have been reported to be cost-effective, clinically useful and can be accessible through the Pan American Health Organization (PAHO) Strategic Fund.^32^, ^33^, ^34^, ^35^ AHD remains a significant challenge, particularly in resource-limited settings where access to diagnostic tools such as CD4 testing is often restricted. While point-of-care CD4 testing offers a more accessible alternative to gold standard flow cytometry, performance issues under uncontrolled conditions can lead to inaccuracies, potentially compromising clinical decision-making. Future studies will evaluate the performance of point of care CD4 tests in real-world settings and access outcomes for patients lost to follow-up, which are crucial for improving AHD management.

In alignment with WHO guidelines,^3^ point-of-care testing for OIs, particularly CrAg testing in serum, is recommended as part of the package of care for patients with AHD. Specifically, CrAg testing is recommended in serum as a screening tool for all patients with CD4 cell counts ≤100 cells/mm^3^ and suggested for those with CD4 cell counts ≤200 cells/mm^3^, irrespective of symptoms. Positive CrAg results warrant pre-emptive antifungal therapy, assuming the absence of central nervous system infection.^2^^,^^36^ TB LAM testing is also recommended for adults and adolescents with HIV who have signs or symptoms, screen positive for TB, or are seriously ill or have AHD. In contrast, there is no WHO recommendation for routine screening of histoplasmosis using *Histoplasma* antigens, as there is insufficient evidence supporting the benefits of antifungal therapy for asymptomatic patients. Since our cohort predominantly consisted of symptomatic hospitalized patients with AHD, the benefit of screening these individuals with point-of-care testing for OIs is clear. A standardized approach using a harmonized threshold for the three diseases could be useful for implementation.

Another important issue is the limited capacity for CD4 cell count testing in many parts of the world. Rapid CD4 testing may help manage such patients, by identifying those who require further investigation for OIs. In this present cohort, over 95% of patients exhibiting any of the inclusion criteria had a CD4 count of <200 cells/mm^3^, consistent with the current HIV epidemic in Brazil, where most patients who are ill enough to require hospitalization have AHD. These patients are likely to benefit the most from the use of point-of-care diagnostic tests to evaluate the presence of OIs.

The study had several limitations. Notably, we lacked information on the frequency of empirical therapy for OIs other than TB. However, it is plausible that the introduction of diagnostic tests in clinical practice reduced the use of empirical therapies due to the high negative predictive value of the assays. Additionally, we were unable to differentiate at study entry the proportion of patients included due to low CD4 counts with limited symptoms. Since the study focused solely on hospitalized patients, this frequency is expected to be minimal, likely negligible. Although a high proportion of patients in the study had previously been exposed to ART, the reasons for ART failure or poor adherence were not evaluated. Another limitation was the lack of information on the treatment of OIs and the absence of diagnosis for other relevant OIs, such as pneumocystosis. A further limitation of our study is the use of 30- and 90-day follow-up periods to assess outcomes. While these timeframes were chosen based on prior studies and the natural history of OIs in advanced HIV, different timeframes might yield different results. For instance, longer follow-up periods could reveal additional late mortality or complications, while shorter periods might not fully capture the impact of early interventions. Future studies should consider evaluating outcomes at multiple time points to better understand the temporal dynamics of OI-related mortality in advanced HIV. Additionally, multivariate analysis was not performed to evaluate independent risk factors for mortality, which is the focus in a separate publication.

Patients from the retrospective and prospective cohorts differed in baseline CD4 count, which might have influenced the frequency of OIs. Although this was a prospective study, some variables had missing data, resulting in different denominators for each evaluation. Many individuals were lost to follow-up, and we were not able to determine the reasons for such failures. Furthermore, these data predominantly reflect the HIV epidemic profile in Porto Alegre, as most patients were included there. This might not reflect OI prevalence in the rest of the country. Challenges associated with the implementation of point-of-care diagnostics, such as logistical constraints, training needs for local staff, and potential resistance from healthcare providers, may also impact on the scalability and sustainability of the initiative.

In conclusion, our study demonstrated the value of incorporating point-of-care tests for early diagnosis of TB, cryptococcosis, and histoplasmosis in hospitalized patients with AHD in Brazil. This resulted in a marked increase in the diagnosis of OIs and facilitated the management of these complex medical conditions.

## Contributors

The manuscript was drafted by AC Pasqualotto and F Perez. All authors contributed substantially to the conception, design, analysis, and interpretation of data, and participated in the preparation and review of drafts of this manuscript and gave their final consent to the publication of the final version. LR, ACP, TV, and FP accessed and verified the raw data in the study.

## Data sharing statement

All data used for this study are available upon successful application to the study team via the corresponding author.

## Declaration of interests

Prof. Pasqualotto has received research grants from Gilead Sciences, Pfizer, MSD, IMMY, MiraVista, and PAHO. He has consulted for Gilead Sciences, Pfizer, MSD, IMMY, and Knight Therapeutics, and given paid talks on behalf of Gilead Sciences, Pfizer, Knight Therapeutics (previously United Medical), MSD, Teva, IMMY, Astellas, Mundipharma, Eurofarma, Sanofi, bioMeriéux, and Sandoz. Dr. Falci has received research grants and consulting fees from Pfizer, MSD, and Gilead Sciences; also reports travel support from Pfizer, United Medical, Janssen, and Merck; participation on Data Safety Monitoring or Advisory Boards for Gilead Sciences, Merck, and GlaxoSmithKline (GSK); has given paid lectures on behalf of Astra-Zeneca, Pfizer, Janssen, GSK, Merck, Gilead Sciences, Knight Pharmaceuticals; and received nonfinancial research support from IMMY. All other authors declare no competing interests.

## Appendix A Supplementary data

Translated Abstract
