# Supplementary material for: Impact of the introduction of a package of care involving early detection of opportunistic infections, a prospective multicenter cohort study of people living with HIV/AIDS in Brazil
Source: Lancet Reg Health Am. 2025 Apr 3;45:101085. doi: 10.1016/j.lana.2025.101085 (PMC11999676; doi:10.1016/j.lana.2025.101085)
Supplement: Translated Abstract [file mmc1.docx]

Editor note: This translation in Portuguese was submitted by the authors and we reproduce it as supplied. It has not been peer reviewed. Our editorial processes have only been applied to the original abstract in English, which should serve as reference for this manuscript.

**Resumo**

**Introdução:** As infecções oportunistas (IOs) são uma das principais causas de morbidade e mortalidade em pessoas com HIV avançado. Este estudo avalia a eficácia dos testes diagnósticos no ponto de atendimento (point-of-care, POC) para tuberculose (TB), histoplasmose e criptococose na rotina de cuidados com HIV no Brasil.

**Métodos:** Foi conduzido um estudo de coorte prospectivo e multicêntrico em cinco hospitais, incluindo pessoas vivendo com HIV (PVHIV) com contagem de células T CD4+ <200 células/mm³ ou com sintomas de IO, independentemente da contagem de CD4, além de pacientes virgens de tratamento, aqueles iniciando terapia e indivíduos com carga viral não suprimida e perda de seguimento (>3 meses). Os testes POC incluíram VISITECT CD4 Advanced Disease, TB LAM Ag (Abbott), GeneXpert MTB/RIF (Cepheid), Histoplasma antigen LFA (MiraVista) e CrAg LFA (IMMY). Os pacientes foram acompanhados por 30 e 90 dias. Dados retrospectivos dos seis meses anteriores ao estudo foram coletados para comparação.

**Resultados:** Entre 419 PVHIV (55% homens cisgênero, 44% mulheres cisgênero, 1% transgênero; idade média: 42 anos, DP ±11,1), 46% apresentaram IOs confirmadas: TB (34%), criptococose (12%) e histoplasmose (10%). As coinfecções foram frequentes, sendo TB e histoplasmose combinadas em 44% dos casos. Meningite criptocócica e histoplasmose grave foram diagnosticadas em 5% e 6%, respectivamente. O teste TB LAM foi positivo em 27% dos casos, sendo 74% classificados como TB disseminada. O uso de testes POC aumentou as taxas de detecção de TB (1,8 vezes), criptococose (2,8 vezes) e histoplasmose (2,8 vezes) em comparação com os dados históricos. As taxas de sobrevivência foram de 87% em 30 dias e 80% em 90 dias, com a antigenemia criptocócica associada a maior mortalidade.

**Interpretação:** O uso de testes POC aprimorou o diagnóstico de IOs, apoiando as diretrizes da OMS. Esses achados destacam a importância da integração de diagnósticos rápidos nos programas de HIV e a necessidade de mais pesquisas sobre os desfechos a longo prazo.

**Patrocínio:** Organização Panamericana de Saúde (OPS)

**Palavras-chave:** Brasil, estudo de coorte, HIV, AIDS, tuberculose, histoplasmose, criptococose, testes rápidos.
